# Ultrasound elastography in the assessment of post-stroke muscle stiffness: a systematic review

**DOI:** 10.1186/s13244-022-01191-x

**Published:** 2022-04-05

**Authors:** Jacqueline Roots, Gabriel S Trajano, Davide Fontanarosa

**Affiliations:** 1 School of Clinical Sciences, Queensland University of Technology, Gardens Point Campus, 2 George St, Brisbane, QLD 4000 Australia; 2 Centre for Biomedical Technologies (CBT), Queensland University of Technology, 2 George St, Brisbane, QLD 4000 Australia; 3 School of Exercise and Nutrition Sciences, Queensland University of Technology, Gardens Point Campus, 2 George St, Brisbane, QLD 4000 Australia

**Keywords:** Ultrasound elastography, Strain elastography, Shear wave elastography, Stroke, Muscle stiffness

## Abstract

**Background:**

Post-stroke muscle stiffness is a major challenge in the rehabilitation of stroke survivors, with no gold standard in clinical assessment. Muscle stiffness is typically evaluated by the Modified Ashworth Scale or the Tardieu Scale; however, these can have low reliability and sensitivity. Ultrasound elastography is an advanced imaging technology that can quantitatively measure the stiffness of a tissue and has been shown to have good construct validity when compared to clinically assessed muscle stiffness and functional motor recovery.

**Objective:**

The purpose of this article is to systematically review the literature regarding the change in muscle stiffness as measured by ultrasound elastography in stroke survivors.

**Methods:**

Scopus, PubMed, Embase, CINAHL, MEDLINE and Cochrane Library were searched for relevant studies that assessed the change in stiffness of post-stroke muscle stiffness measured by ultrasound elastography following the Preferred Reporting Items for Systematic Reviews and Meta-Analysis (PRISMA) guidelines.

**Results:**

In total, 29 articles were identified, using either strain elastography and shear wave elastography to measure the stiffness of muscles in stroke survivors, most frequently in the biceps and medial gastrocnemius muscles. The stiffness was typically higher in the paretic compared to the non-paretic or healthy control. Other variations that increased the stiffness include increasing the joint angle and introducing a passive stretch or muscle activation. The paretic muscle has also been assessed pre- and post-treatment demonstrating a decrease in stiffness.

**Conclusion:**

Ultrasound elastography is a promising imaging technology for determining the muscle stiffness in stroke survivors with need for a standardized imaging protocol.

## Key points

- Ultrasound elastography can assess the change in muscle stiffness of stroke survivors.
- Paretic limbs display higher stiffness than non-paretic limbs.
- Altering the joint angle will alter the muscle stiffness.
- Ultrasound elastography stiffness correlated with clinical assessments most of the time.
- Muscle stiffness decreased after rehabilitation or pharmacological treatment.

## Introduction

Advancements in the treatment of stroke have resulted in an increased survival rate; however, the likelihood of developing spasticity remains unchanged [1, 2]. Spasticity is a common complication of stroke management which has been defined by Pandyan et al. [3] in the Support Program of Assembly of a Database for Spasticity (SPASM) Consortium as “disordered sensori-motor control, resulting from an upper motor neuron lesion, presenting as intermittent or sustained involuntary activation of muscles.” The impact on daily life from spasticity can range anywhere from sensations of stiffness, heaviness and pain, to problems in functional capability, deformities and pressure sores [4, 5].

Following a stroke, the upper motor neuron lesion may result in alterations of the neural input to muscles and then non-neural mechanical alterations to muscle as a secondary consequence. The response of a muscle to a stretch is influenced by the speed of the stretch, whereby when stretched at low velocities the muscle resistance is generated by the passive elastic properties [4]. After a threshold velocity, a stretch reflex is evoked, and the muscle resistance and stiffness will increase. The non-neural alterations such as changes in muscle, tendon and extracellular matrix composition and joint position can occur as a result of the neurological changes as well as the disuse associated with paralysis or paresis.

There are no clear indicators as to the degree of spasticity that may occur; however, severe paresis in the subacute stage post-stroke does increase the likelihood [6–9]. The development of spasticity occurs in a flexor pattern, affecting the antigravity muscles [6]. In the upper limb, this involves shoulder adduction, forearm pronation and elbow, wrist and finger flexion [10]. In the lower limb, this involves hip adduction, knee extension, ankle plantar flexion and pes varus [10]. There are no differences in rates of occurrence of spasticity with age or sex [8]. Detection and measurement of spasticity is critical to help deliver timely therapy and avoid progression of the disease.

Current clinical assessments of spasticity typically include the Modified Ashworth Scale (MAS) or the Tardieu Scale (TS), both of which are ordinal scales. These assessments measure the resistance to a passive movement and/or the angle of the catch where there is a distinct brief resistance, perceived subjectively by the physician [4, 10, 11]. In the MAS, the limb is passively stretched and the resistance perceived by the physician is scored [12]. For the TS assessment, the limb is passively stretched at a slow velocity to determine soft tissue resistance and again at a fast velocity to elicit the stretch reflex and measure the catch [12, 13].

The clinical assessments do not directly measure spasticity, as they cannot differentiate the neural hypertonicity from the non-neural mechanical characteristics of the limb [4, 11]. The assessments are articular, in that it is not possible to distinguish which specific muscle the resistance is from, or the amount of input from tendons and joints [14]. The MAS and TS are qualitative and subjective [15] and known to have poor inter-rater reliability [7, 15, 16]. A reliable and quantitative assessment of muscle stiffness would allow therapists to better target the muscles that require treatment after a stroke and improve the quality of life for stroke survivors.

Ultrasound elastography is an advanced imaging technique that assesses the stiffness of tissue that has been used clinically in the evaluation of liver, breast, thyroid and prostate mechanical properties [17, 18]. Both strain and shear wave elastography (SWE) are emerging categories of elastography for the musculoskeletal system; however, the method of determining the stiffness varies greatly [19]. Strain elastography (also known as compression elastography) observes and measures the deformation of a tissue when a compressive force is applied, most commonly a light repetitive motion from the operator [20]. The applied stress is not quantifiable; therefore, a semi-quantitative measurement of the strain in the tissue, compared to the strain of a reference tissue (such as the subcutaneous layer), can provide a strain ratio [21]. SWE is a type of dynamic elastography which produces a stress on the tissues by a push beam of focused ultrasound called an acoustic radiation force impulse (ARFI) [21]. This generates shear waves within the medium that travel perpendicular to the ARFI and the propagation velocity is calculated [19]. This velocity is either directly reported in m/s as the shear wave velocity (SWV) or in kPa as the shear modulus as a measure of elasticity [22].

The increasing understanding of ultrasound elastography in healthy muscles has allowed research to progress to the assessment of muscle stiffness in pathological conditions. Benefits of using elastography in post-stroke muscles include detecting changes, quantifying the severity of disease and commenting on the change in stiffness after treatments. Zúñiga et al. [23] recently systematically reviewed the rigor of the methodology when using ultrasound elastography in the assessment of muscle spasticity as a result of stroke, cerebral palsy, multiple sclerosis. A literature review by Lehoux et al. [14] examined the use of SWE for determining muscle stiffness in stroke survivors. The systematic review and meta-analysis by Miller et al. [24] analyzed the reliability and validity of studies that assessed the stiffness of muscles in people with neurological conditions. There have been at least two studies published since these systematic and literature reviews took place. This systematic review aims to evaluate the current literature regarding changes in muscle stiffness as measured by ultrasound elastography in stroke survivors.

## Materials and methods

The databases Scopus, PubMed, Embase, CINAHL, MEDLINE and Cochrane Library were searched between October 2020 and October 2021. The following search terms: (‘stroke’ or ‘poststroke’ or ‘post-stroke’ or ‘cerebrovascular AND accident’) AND (‘spastic*’ or ‘hypertonia’ or ‘stiffness’) AND (‘ultrasound’ or ‘elastography’ or ‘shear wave elastography’ or ‘shearwave’ or ‘sonoelastography’ or ‘strain imaging’ or ‘supersonic’) were used with Boolean operators. Abstracts were screened for inclusion and duplicates were removed. Selection of studies was based on: (1) articles published in English; (2) human populations in vivo; (3) use of ultrasound elastography on muscles for stiffness; (4) participants were stroke survivors. Conference papers and proceedings have been included in this review. Exclusion criteria included: (1) cadaveric studies; (2) published in languages other than English. Backward searching through reference lists of the selected articles and forward searching through cite searches were performed to identify other publications. This review was conducted by a single reviewer (J.R.) following the Preferred Reporting Items for Systematic Reviews and Meta-Analysis (PRISMA) guidelines [25] and prospectively registered with PROSPERO (CRD42020203440). The study methodological characteristics such as sample size, muscles assessed, machine used, joint position, level of muscle activity were recorded, as well as the results.

## Results

**Fig. 1:**
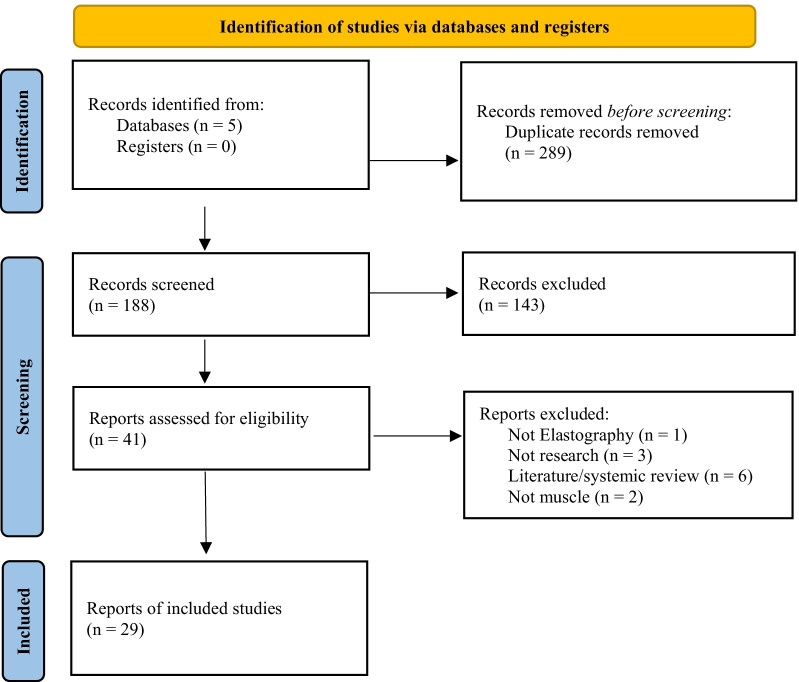
PRISMA flowchart for the identification of studies

Initial searches returned 476 results which were assessed for eligibility. After removing duplicates and screening for relevance, 29 articles were included in this review (Fig. 1). There have been 8 articles published regarding muscle stiffness post-stroke as assessed by strain elastography, 20 using SWE, and one study using both strain and SWE.

### Methodological differences

It is important to note the methodological differences in the studies mentioned as this can have an impact on the results. Table 1 shows the study characteristics. The sample size in the studies ranged from 1 participant [26] through to 60 participants [27]. There was often a minimum time since stroke required for participation. Many studies included participants from at least 6 months after stroke onset, and the earliest was set by Eby et al. [28] involving participants at least 3 months after stroke. Wu et al. [29] and Liu et al. [27] were the only studies to limit the maximum time since stroke, which was set at 6 months. Otherwise, the time since stroke for participants varied widely within each study and was often not limited.

**Table 1:** Methodological characteristics of studies

Author	Elastography	Title	Muscle	Contraction	Participants	System	Transducer	Measured
Lee et al. 2014 [42]	SWE	Measurement of Altered Muscle Properties in Stroke-impaired Muscle Using Shear Wave Ultrasound Elastography	*	*	16	*	*	SWV (m/s)
Kesikburun et al. 2015 [34]	Strain	Assessment of Spasticity With Sonoelastography Following Stroke: A Feasibility Study	GM GL	Passive rest	26	GE Logiq S7	5–12 Linear	Elasticity Index and Ratio
Lee et al. 2015 [48]	SWE	Quantifying changes in material properties of stroke-impaired muscle	BB	Passive rest with EMG	16	SSI Aixplorer	4–15 SuperLinear	SWV (m/s)
Mathevon et al. 2015 [50]	SWE	Reliability of 2D ultrasound imaging associated with transient ShearWave Elastography method to analyze spastic gastrocnemius medialis muscle architecture and viscoelastic properties	GM	Passive rest and at maximal passive stretch by goniometer	14	SSI Aixplorer	*	Shear modulus (kPa)
Park & Kwon 2015 [35]	Strain	Sonoelastographic Findings According to Spasticity of Elbow Flexor in Post-Stroke Hemiplegia	BB and Brachialis	*	19	*	*	Median red, blue and hue pixel intensity of histograms
Eby et al. 2016 [28]	SWE	Quantitative Evaluation of Passive Muscle Stiffness in Chronic Stroke	BB	Passive extension with EMG	9P 4C	Verasonics	L7-4 Philips	Shear modulus (kPa)
Yaşar et al. 2016 [32]	Strain	Assessment of forearm muscle spasticity with sonoelastography in patients with stroke	FDS FDP FCR FCU	Passive rest	23	GE Logiq S7	5–12 Linear	Elasticity Index and ratio
Rasool et al. 2016 [54]	SWE	Altered Viscoelastic Properties of Stroke-Affected Muscles Estimated Using Ultrasound Shear Waves – Preliminary Data	BB	Passive with EMG	3	SSI Aixplorer	4–15 SuperLinear	SWV (m/s)
Jakubowski et al. 2017 [37]	SWE	Passive material properties of stroke-impaired plantarflexor and dorsiflexor muscles	GM TA	Passive rest and passive stretch by dynamometer	14	SSI Aixplorer	4–15 SuperLinear	SWV (m/s)
Wu et al. 2017 [29]	SWE	Evaluation of Post-stroke Spastic Muscle Stiffness using Shear Wave Ultrasound Elastography	BB	Passive with EMG	31P 21C	Siemens Acuson S2000	9L4	SWV (m/s)
Aşkın et al. 2017 [55]	Strain	Strain sonoelastographic evaluation of biceps muscle intrinsic stiffness after botulinum toxin-A injection	BB	Passive	48	Toshiba Aplio 500	8–12 Linear	Strain Index
Eby et al. 2017 [26]	SWE	Quantifying spasticity in individual muscles using shear wave elastography	BB & Brachialis	Passive extension by dynamometer	1	Verasonics	L7-4 Philips	SWV (m/s)
Gao, Chen, et al. 2018 [43]	Strain	Ultrasound Strain Imaging to Assess the Biceps Brachii Muscle in Chronic Poststroke Spasticity	BB	Passive rest and passive extension	7P 8C	Siemens Acuson S3000	9L4	Strain Ratio
Gao, He, et al. 2018 [44]	SWE	Quantitative Ultrasound Imaging to Assess the Biceps Brachii Muscle in Chronic Post-Stroke Spasticity: Preliminary Observation	BB	Passive	7P 8C	Siemens Acuson S3000	9L4	SWV (m/s)
Hong et al. 2018 [30]	Strain	Quantitative Evaluation of Post-stroke Spasticity Using Neurophysiological and Radiological Tools: A Pilot Study	GM	Passive	8	GE Logiq S9	9–15 Linear	Elasticity Index
Mathevon et al. 2018 [38]	SWE	Two-Dimensional and Shear Wave Elastography Ultrasound: A Reliable Method to Analyse Spastic Muscles?	GM TA	Passive	14	SSI Aixplorer	4–15 SuperLinear	Shear modulus (kPa)
Rasool et al. 2018 [49]	SWE	Shear Waves Reveal Viscoelastic Changes in Skeletal Muscles After Hemispheric Stroke	BB	Passive with EMG	13	SSI Aixplorer	4–15 SuperLinear	SWV (m/s)
Saadat et al. 2018 [45]	SWE	Frequency Dependence of Shear Wave Velocity in Stroke-Affected Muscles During Isometric Contraction- Preliminary Data	BB	Isometric contraction with EMG	3	SSI Aixplorer	4–15 SuperLinear	SWV (m/s)
Galvão et al. 2019 [33]	SWE	Quantitative Analysis of Intrinsic Muscle Stiffness in Biceps Brachii of Post-stroke Patients	BB	Passive	10	SSI Aixplorer	SL10-2	Shear modulus (kPa)
Gao et al. 2019 [56]	SWE and Strain	Ultrasound Elastography to Assess Botulinum Toxin A Treatment for Post-Stroke Spasticity: A Feasibility Study	BB	Passive	7	Siemens Acuson S3000	9L4	SWV (m/s) and strain ratio
Lee et al. 2019 [47]	SWE	Muscle material properties in passive and active stroke-impaired muscle	BB	Isometric contraction with EMG	14P 8 C	SSI Aixplorer	*	SWV (m/s)
Le Sant et al. 2019 [40]	SWE	Effects of stroke injury on the shear modulus of the lower leg muscle during passive dorsiflexion	GM GL SOL FDL FHL TA EDL	Passive stretch with EMG	14P 13 C	SSI Aixplorer	SL10-2 or SL15-4	Shear modulus (kPa)
Leng et al. 2019 [36]	SWE	Alterations of Elastic Property of Spastic Muscle With Its Joint Resistance Evaluated From Shear Wave Elastography and Biomechanical Model	FCR	Passive stretch with EMG	15	SSI Aixplorer	4–15 SuperLinear	Shear modulus (kPa)
Liu et al. 2020 [27]	SWE	The Value of Real-Time Shear Wave Elastography before and after Rehabilitation of Upper Limb Spasm in Stroke Patients	BB	Passive	60	GE Logiq E9	9L	SWV (m/s) and Young’s Modulus
Huang et al. 2020 [39]	SWE	Whole-body vibration modulates leg muscle reflex and blood perfusion among people with chronic stroke: a randomized controlled crossover trial	GM SOL	Passive	36	SSI Aixplorer	4–15 SuperLinear	Shear modulus (kPa)
Analan & Ozdemir 2020 [51]	SWE	Assessment of Post-Stroke Biceps Brachialis Muscle Stiffness by Shear-Wave Elastography: a Pilot Study	BB	Passive	24	Siemens Acuson S2000	L9-4	SWV (m/s)
Furukawa & Masakado 2021 [41]	Strain	Changes in Sonoelastography After Using Botulinum Toxin Type A for the Treatment of the Patients with Post-stroke Spasticity: Report of 2 Cases	BB GM	Passive	2	Hi Vision Ascendus, Hitachi Aloka Medical	EUP-L65 6-14 MHz	Strain Ratio
Yoldaş Aslan et al. 2021 [31]	Strain	Does extracorporeal shock wave therapy decrease spasticity of ankle plantar flexor muscles in patients with stroke: A randomized controlled trial	G	Passive	17 Act 17 Sh 17 C	GE Logiq S7	ML6-15	Strain Index
Liu et al. 2021 [46]	SWE	Quantitative Ultrasound Texture Analysis to Assess the Spastic Muscles in Stroke Patients	BB	Passive	22	Siemens Acuson S2000	L9-4	SWV (m/s)

SWE, shear wave elastography; SWV, shear wave velocity; GM, gastrocnemius medialis; GL, gastrocnemius lateralis; BB, biceps brachii; TA, tibialis anterior; SOL, soleal; FCR, flexor carpi radialis; FDS, flexor digitorum superficialis; FDP, flexor digitorum profundus; FCU, flexor carpi ulnaris; FDL, flexor digitorum longus; FHL, flexor hallucis longus; EDL, extensor digitorum longus; G, gastrocnemius; EMG, electromyography; P, paretic; C, control; Act, Active; Sh, sham

While Hong et al. [30] and Yoldaş Aslan et al. [31] included patients with an MAS or TS score of at least 1, most other authors did not have a spasticity criteria for their inclusion of participants. Yaşar et al. [32], Galvão et al. [33] and Kesikburun et al. [34] had the greatest spasticity requirements for inclusion, requiring an MAS of ≥ 1 + . Wu et al. [29] included only participants with a normal elbow range of motion.

The biceps brachii was assessed most frequently (*n* = 14), with other upper limb muscles investigated including the brachialis [26, 35], flexor digitorum superficialis, flexor digitorum profundus, flexor carpi radialis [36], and flexor carpi ulnaris [32]. The assessment of lower limb muscles has primarily been on the medial gastrocnemius muscle (*n* = 8), though the lateral gastrocnemius, tibialis anterior [37, 38], soleus [39], flexor digitorum longus, flexor hallucis longus, extensor digitorum longus [40] have been investigated as well. Yoldaş Aslan et al. [31] assessed the gastrocnemius and however did not differentiate the medial and lateral muscles. The study by Furukawa and Masakado [41] investigated the muscles of both the upper and lower limbs, with two cases of concurrent biceps and medial gastrocnemius spasticity.

The variability in patient positioning is another factor to consider as a change in muscle position can alter the passive stiffness. The most frequent positioning for the biceps was in a seated position, with the forearm supported, and the wrist in a neutral position. A few authors commented on the specific abduction and flexion of the shoulder, and some did not comment on the positioning of the participant at all [34, 35, 42].

The SuperSonic Imagine Aixplorer machine was used in the majority of studies (*n* = 12); however, the GE Logiq S7, Logiq S9, Logiq E9, Toshiba Aplio 500, Verasonics, Hitachi Hi Vision Ascendus, Siemens Acuson S2000 and Acuson S3000 were also used. The machine used was not stated in two studies [35, 42]. Where mentioned, a high-frequency linear transducer was selected, typically 4–15 MHz.

Surface electromyography (sEMG) can be used to monitor electrical activity of the muscles during assessment. Nine studies used sEMG to confirm that muscles were assessed at rest or to visually control the level of voluntary contraction. Other passive muscle assessments have relied on subjective clinical judgment from the ultrasound machine operator.

Gao, Chen et al. [43] used passive extension and flexion of the biceps muscle to create the deformation in their longitudinal strain. This was achieved by using speckle tracking to compare the lengthening and shortening of the muscle in the movement. Their axial strain deformation was created by attaching a 1-kg sandbag to the transducer with the difference in anteroposterior length measured. All other strain elastography studies in this review used manual compression techniques to create the deformation.

### Reported findings

#### Paretic versus non-paretic

The comparison of the paretic muscle to the non-paretic muscle was the most common method to determine whether there was a change in muscle stiffness after a stroke. The muscle stiffness was typically increased when compared to the non-paretic side [26, 29, 30, 32, 34, 37, 42–48]. Rasool et al. [49] noted an increased stiffness of the stroke-affected biceps compared to the non-affected at 150° and 120° elbow flexion; however, no difference to the non-affected side at 90° flexion was noticed.

There were three instances in the literature that reported a small decrease in the stiffness of the paretic muscle. The shear modulus of the paretic muscle was decreased in the study by Mathevon et al. [50] and in the subgroup analysis of nine participants with an MAS of 0 in the study by Wu et al. [29] but only in the 90° elbow position, not the fully extended elbow. Le Sant et al. [40] found a decreased shear modulus in the tibialis anterior and extensor digitorum longus in stroke survivors at 80% of their range of motion compared to healthy controls. However, they did find an increased shear modulus of the medial and lateral gastrocnemius muscles in stroke survivors, suggesting that gastrocnemius muscles may be preferentially affected by spasticity after a stroke. The study by Analan & Ozdemir [51] and Huang et al. [39] assessed the muscles of stroke survivors and did not find any statistically significant difference between the paretic and the non-paretic limbs.

#### Paretic versus healthy controls

The stiffness of paretic muscles was compared to muscles of healthy controls, with no history of stroke in 6 studies. All six studies found an increased stiffness in the paretic muscle compared to healthy controls [28, 29, 40, 43, 44, 47]. It is noted that Gao, He et al. [43] only found this difference when the elbow was in full extension.

#### Altering joint angles

**Fig. 2:**
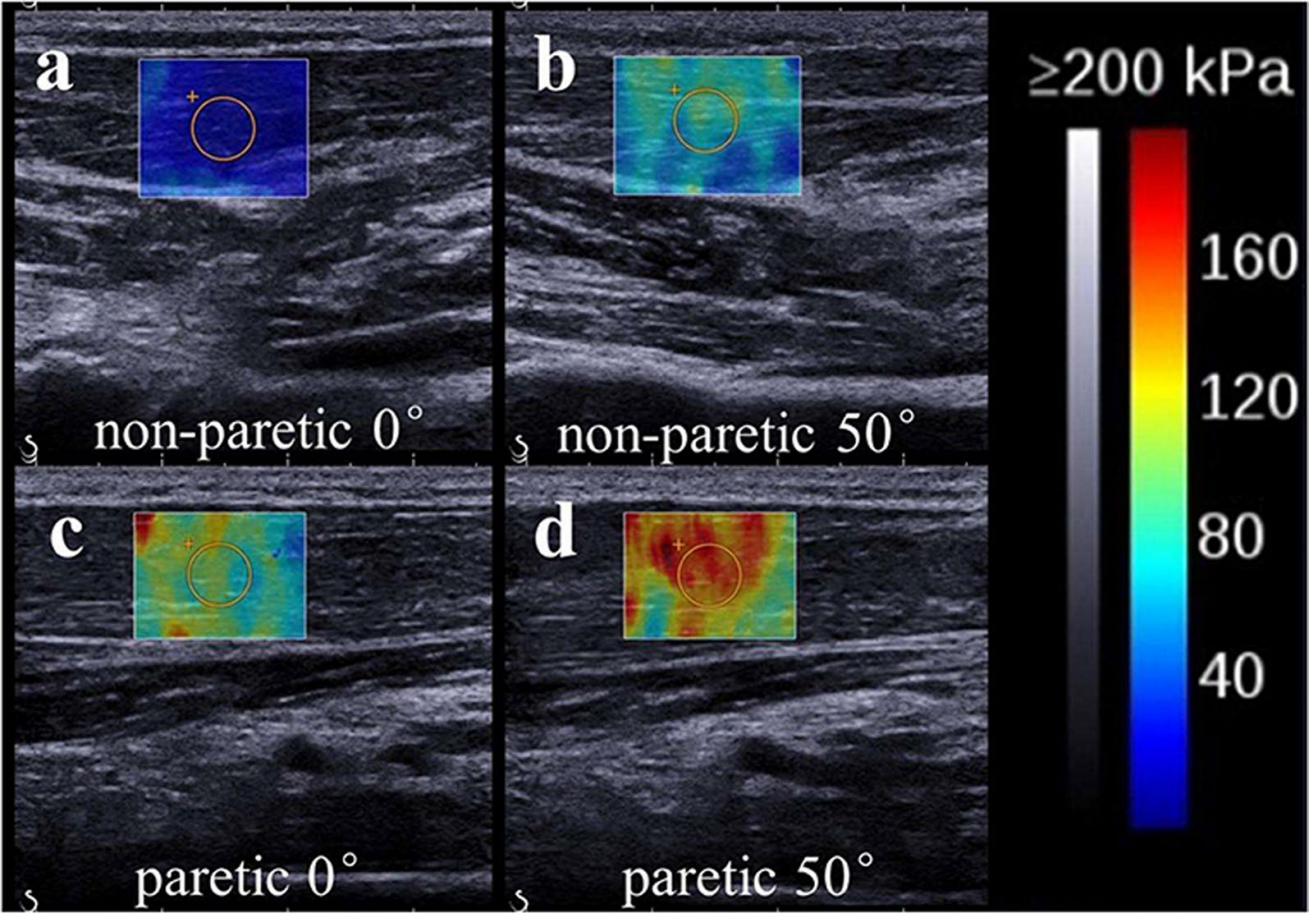
SWE superimposed on the B-mode ultrasound image of the muscles of the (**a**) neutral non-paretic, (**b**) stretched non-paretic, (**c**) neutral paretic and (**d**) stretched paretic forearm. From Leng et al. (2019) [36]

The angle of the joint can influence the stiffness of the muscles. The passive paretic biceps were assessed in different elbow positions in 4 studies, each finding an increased muscle stiffness with increased elbow extension with Wu et al. [29] and Gao, He et al. [44] recording at full extension and 90° elbow flexion, while Eby et al. [28] recorded at 80° and 150° elbow extension and Rasool et al. [49] recorded at 90°, 120° and 150° elbow extension. The passive movement of the wrist from 0° palmar flexion to 50° dorsal flexion to stretch the muscle also increased the stiffness of the flexor carpi radialis of stroke survivors (Fig. 2) [36]. Comparably, the change from plantar flexion to dorsiflexion in the ankle by passive movement increased the SWV of the medial gastrocnemius in the study by Jakubowski et al. [37].

#### Passive stretch

Eby et al. [28] assessed the biceps in a passive stretch, controlled by a dynamometer at 5° per second, 20° per second and 40° per second. The high-speed image acquisition times of the SWE machine allowed them to assume the muscle was static. They found that approximately half of the stroke survivors had SWV which correlated with torque, and that the stiffness increased as angle and the velocity increased. Eby et al. [26] performed similar assessments on a single participant at speeds of 5° per second, 20° per second, 40° per second and 60° per second between 105° and 165° and found an increased stiffness compared to the non-affected side at all velocities in both the biceps and the brachialis muscle. Other passive stretch studies include Mathevon et al. [50] and Jakubowski et al. [37] in their assessments of the medial gastrocnemius at rest and at a maximal passive stretch by a goniometer or dynamometer. Mathevon et al. [50] did not comment on the change stiffness as the stretch occurred, but analyzed that the coefficient of variation of the paretic muscle increased in the stretched state. Jakubowski et al. [37] reported that the SWV of the medial gastrocnemius increased from plantar flexion to dorsiflexion at a greater rate on the paretic side compared to the non-paretic side.

#### Active state

To assess the paretic muscle in an active state, Saadat et al. [45] assessed the biceps under isometric contraction of 0%, 10%, 20% and 30% of the maximum voluntary contraction (MVC). They found that while in an active state, the SWV was higher on the non-paretic side compared to the paretic. Lee et al. [47] also assessed the biceps under active isometric elbow contraction at 0%, 10%, 25%, 50%, 75% and 100% of the MVC. They found that the slope of SWV versus activation level was lower in paretic sides compared to both the non-paretic and the healthy controls.

#### Correlations with clinical assessments

The correlation between ultrasound elastography stiffness and clinical assessments was not a primary aim of any of the studies; however, some authors attempted to analyze the relationship. The MAS and TS have been frequently used as a scale to grade the spasticity. The Fugl-Meyer Assessment, used for physical independence and capability [52], and the Brunnstrom Motor Recovery Stages, used for neurological recovery [53], were also used as clinical assessments. The MAS correlated with ultrasound elastography stiffness in 10 studies and did not correlate in 4 studies. The TS correlated with elastography stiffness in all 3 studies that commented on correlation. The Fugl-Meyer Assessment correlated with muscle stiffness in 3 studies and did not correlate in 1 study [36]. The Brunnstrom Motor Recovery Stages did not correlate with SWV in the study by Analan and Ozdemir [51].

#### Phase versus group velocities

SWE provides a measurement of the elasticity of the tissue and makes the assumption that the viscosity is negligible. The SWV averaged over all velocities, has been coined as group velocities, and the frequency-dependent SWV is termed phase velocity. The use of SWE by most authors has been of group velocities; however, this has been investigated. Rasool et al. [54] assessed the post-stroke biceps muscle stiffness with both a group velocity and five selected phase velocities and compared the results to the non-paretic limb. They found that there were differences in the returned SWV at varying phase velocities, and that this difference was more pronounced in the paretic limb. Building on their previous work, Rasool et al. [49] assessed the group velocities and phase velocities to estimate the elastic and viscous moduli using a Voigt model. They again found that the dispersion of phase velocities differs from the group velocity. Saadat et al. [45] also investigated the phase velocities in the paretic and non-paretic biceps and agreed that there was a difference between the dispersion of the group and phase velocities. This demonstrates that the muscle tissue displays both elastic and viscous properties, rather than negligible viscous properties, as assumed by most other SWE investigations. Despite the difference in the measurement of the SWV, all three authors found an increased group velocity in the paretic limb compared to the non-paretic.

#### Changes in muscle stiffness over time

There were no studies that specifically assessed for changes over time; however, a few authors attempted to correlate the time since stroke with the muscle stiffness. Wu et al. [29] found a positive correlation between the time since stroke and the SWV within the biceps at 90° elbow flexion, but not at 0° flexion. Lee et al. [48] observed a correlation between the length of time since stroke and the difference in SWV between the participants’ paretic and non-paretic sides. Aşkın et al. [55] and Analan and Ozdemir [51] did not find any correlation between time since stroke and the strain index or SWV, respectively. In an assessment of intrasession reliability, Mathevon et al. [50] assessed the stiffness of stroke survivors’ gastrocnemius medialis by SWE over two sessions, 7 days apart. There were no longitudinal assessments of post-stroke spasticity without treatment found within the literature for either strain elastography or SWE.

#### Changes in muscle stiffness after treatment

The objectives of therapy and antispasticity medications include reducing the degree of spasticity, improving joint function and decreasing pain. Rehabilitation therapy is highly patient-specific and requires daily commitment. Botulinum Toxin-A (BoNT-A) injections are administered into the muscle by the physician as a relaxant treatment to reduce muscle activation [41]. The therapeutic response from these options, much like the initial development of spasticity, is measured by clinical tests such as the MAS and TS; however, there is growing literature to support the use of ultrasound elastography to measure the response.

The effect of BoNT-A injections into the biceps muscle was evaluated by Aşkın et al. [55] by strain elastography. Their research showed a decreased strain ratio (correlating with less stiff muscles) at 4 weeks post-injection. Gao et al. [56] also assessed the change in muscle stiffness after BoNT-A by both SWE and strain elastography. All patients (*n* = 7) were evaluated again between 17 and 30 days after injection, and there was a decrease in SWV from 1.90 to 1.63 m/s at 90° elbow flexion suggesting reduced muscle stiffness. More recently, Furukawa and Masakado [41] investigated the change in strain ratio of both the biceps and medial gastrocnemius before and at 2, 4, 8 and 12 weeks after a BoNT-A injection. They noted a decrease in muscle stiffness; however, the change for each of their two participants followed a different pattern.

Liu et al. [27] measured the SWV in spastic muscles before and immediately after therapy and functional electrical stimulation. The spastic muscles had a significant reduction in SWV from the treatment, despite having a higher SWV compared to the non-affected side both pre- and post-therapy.

Huang et al. [39] explored the effects of whole-body vibration on the shear modulus of the medial gastrocnemius muscle every minute for the first five minutes after vibration. They did not find any difference in the stiffness of the paretic or non-paretic muscles from this intervention.

Yoldaş Aslan et al. [31] assessed the effect of extracorporeal shock wave therapy on the stiffness of the gastrocnemius muscle measured immediately prior, after the 2 week therapy and 4 weeks later. This randomized controlled trial also included a sham therapy and a control group. The strain index decreased in all three groups; however, the clinical assessments only decreased in the extracorporeal shock wave therapy group. Given that there were no between-group differences, they interpreted that the conventional therapy received by all groups was the reason for the decreased strain index.

## Limitations of the studies

The passive state of muscles was confirmed by sEMG in nine studies which allows for a greater confidence that there was no contraction. Studies that did not use sEMG cannot rule out the possibility that there was underlying muscle contraction, which would increase the stiffness of the muscle.

The ultrasound elastography systems have a maximum measurable velocity and any velocities higher than this are only recorded as being at the limit. Therefore, if the true velocity was higher than the machine maximum, it would be underreported. This is not a concern for the studies that assess the muscle in a passive state; however, this is a limitation in the active studies. Lee et al. [47] were limited to 16 m/s, which they found to be approximately 70% of the subjects’ MVC.

The transducer pressure applied by the operator during both shear wave and strain elastography can deform and affect the deeper structures. An increased amount of transducer pressure would result in altered values for strain elastography and higher velocities in SWV. Similarly, an unequal pressure along the length of the transducer will create disproportionate measurements within the elastogram.

The experience level of the elastography performer was not mentioned in every study. Ultrasound and, by extension, elastography are highly operator dependant, and the level of experience of the operator can have an impact on the results.

One major consideration is the time since stroke of the participants in the studies. The earliest assessment of spasticity post-stroke was performed by Wu et al. [29] where the duration from the stroke onset was less than 3 months in 93% of their participants (*n* = 29/31). Liu et al. [27] set an inclusion criterion of participants who were between the first- and sixth-months post-stroke. No studies of muscle stiffness assessed by ultrasound elastography in the acute stage post-stroke were found; therefore, it is difficult to extrapolate the findings to early-stage post-stroke survivors.

## Discussion

This systematic review has appraised the literature regarding ultrasound elastography in the assessment of muscle stiffness after a stroke. The studies of SWE in post-stroke muscle stiffness have substantial methodological differences such as machine, body/joint position and active/passive state which should be highlighted. The image acquisition protocol should be described and maintained throughout the assessment with the intent that future researchers can replicate the protocol. There were no overarching guidelines or protocols followed, nor found within the literature. All these factors contribute to the variation in results and must be considered when attempting to generalize findings.

The inclusion and exclusion criteria are the leading varying factors with some studies limiting the severity of spasticity to those with an MAS score equal or greater than 1. The studies by Yaşar et al. [32], Galvão et al. [33], Gao, Chen et al. [43] and Kesikburun et al. [34] included participants with an MAS ≥ 1 + . An inclusion of participants with clinical assessments that indicate a higher degree of stiffness would likely result in findings of higher stiffness.

The stiffness of the muscles was increased compared to the paretic side in most of the studies which is suggestive of an alteration in the elastic properties. However, Mathevon et al. [50] and the MAS = 0 subgroup analysis by Wu et al. [29] found a decreased stiffness. The decrease of the SWV in the subgroup is theorized to be because of the flaccidity in the acute phase. The decreased stiffness found in the tibialis anterior and extensor digitorum longus by Le Sant et al. [40] may be a reflection of the muscle-specific changes that typically affect the flexor muscles. There were two studies which found no difference in the stiffness between the paretic and non-paretic limbs [39, 51]. The reason for these mixed results is not evident but encourages more research to evaluate the correct use of ultrasound elastography in the assessment of post-stroke muscle stiffness.

Similar to assessments made in healthy muscles the stiffness of paretic muscles is affected by the joint angle and the level of activation. The increased elbow extension increased the stiffness of the biceps [26, 29, 44]. The change from palmar flexion to dorsiflexion increased the stiffness of the flexor carpi radialis [36] as did the change from plantar flexion to dorsiflexion for the gastrocnemius medialis [37]. In assessments of active muscle contraction, Saadat et al. [45] and Lee et al. [47] found an increased SWV in the active state compared to the passive state.

Clinical implications of detecting changes in muscle stiffness include potentially establishing a more accurate assessment for spasticity; however, research correlating clinical assessments of muscle stiffness with ultrasound elastography measurements is still in infancy. Most studies have commented on the correlation with clinical assessments, but the generally low sample sizes do limit evaluations. Despite no studies having a primary aim of correlating clinical assessments with muscle stiffness measured by ultrasound elastography, there were multiple attempts made in most of which the correlation proved true. Congruently, the recent meta-analysis by Miller et al. [24] found that there was a positive correlation between the SWV values and the MAS and TS on the paretic side.

The assessment of muscle mechanical properties by strain elastography and SWE can be used to comment on the effect of the pharmacological or physical therapies. The literature supports findings of decreased stiffness which is in line with the aims of treatments such as BoNT-A injections and shock wave therapy. This demonstrates the potential for ultrasound elastography to be used not only in categorizing the changes in muscle stiffness in post-stroke, but also for quantifying the effect of treatment and potentially guiding the treatment to increase the efficacy.

An early assessment of the muscle mechanical properties before the non-neural components begin to take effect would be, in our opinion, the most appropriate ‘base-line’ value. Wu et al. [29] had the earliest inclusion criteria of less than 6 months and found a correlation between the SWV of the biceps in a longitudinal axis and the duration of time since stroke. More research is needed to determine the stiffness of muscles as measured by ultrasound elastography in the acute stage post-stroke.

In particular, a longitudinal assessment of post-stroke muscle stiffness would help diagnose spasticity development or predict the benefit of therapy. A longitudinal assessment would provide a patient-specific, directly comparable result. The many variables for SWE and the dynamic nature of muscle may be, at least in part, overcome by comparing a patient’s SWV with their SWV at a different point in time, rather than comparing them with a clinical standard. This is especially logical since the literature review has shown that there is still a lot of discrepancy in what can be considered an acceptable clinical standard.

## Conclusions

Ultrasound elastography to assess muscle stiffness in the post-stroke population has overt clinical applications. This systematic review found that the paretic limb typically had an increased stiffness compared to the non-paretic as well as to control participants. The muscle stiffness increased under a variety of conditions such as altering the joint angle, the level of rest and contraction, and the speed of dynamic movement. The correlation between the current clinical assessments and ultrasound elastography is promising, and these advanced technologies could provide an excellent quantitative and reliable alternative.

Despite this, there are many facets of research surrounding ultrasound elastography in post-stroke muscles which remain untouched. There appears to be gaps in the literature regarding the assessment of muscle stiffness in the acute stage after a stroke, which would give an understanding of the development of spasticity from a baseline assessment. Another gap includes a longitudinal assessment, which would be integral in determining the patient-specific development of stiffness. A longitudinal assessment of the change in muscle stiffness in the acute stage post-stroke would address the gap in the research and provide therapists with valuable information about the development of non-neural components of spasticity.

## Data Availability

Not applicable.
